# The zinc spark is an inorganic signature of human egg activation

**DOI:** 10.1038/srep24737

**Published:** 2016-04-26

**Authors:** Francesca E. Duncan, Emily L. Que, Nan Zhang, Eve C. Feinberg, Thomas V. O’Halloran, Teresa K. Woodruff

**Affiliations:** 1 Department of Obstetrics and Gynecology, Feinberg School of Medicine, Northwestern University, Chicago, IL 60611, USA; 2 The Chemistry of Life Processes Institute, Northwestern University, Evanston, IL 60208, USA; 3 Fertility Centers of Illinois, Chicago, IL 60610, USA; 4 Department of Chemistry and Department of Molecular Biosciences, Northwestern University, Evanston, IL 60208, USA

## Abstract

Egg activation refers to events required for transition of a gamete into an embryo, including establishment of the polyspermy block, completion of meiosis, entry into mitosis, selective recruitment and degradation of maternal mRNA, and pronuclear development. Here we show that zinc fluxes accompany human egg activation. We monitored calcium and zinc dynamics in individual human eggs using selective fluorophores following activation with calcium-ionomycin, ionomycin, or hPLCζ cRNA microinjection. These egg activation methods, as expected, induced rises in intracellular calcium levels and also triggered the coordinated release of zinc into the extracellular space in a prominent “zinc spark.” The ability of the gamete to mount a zinc spark response was meiotic-stage dependent. Moreover, chelation of intracellular zinc alone was sufficient to induce cell cycle resumption and transition of a meiotic cell into a mitotic one. Together, these results demonstrate critical functions for zinc dynamics and establish the zinc spark as an extracellular marker of early human development.

A critical window of early development is egg activation, a sequence of events that marks the transition of a mature egg into a developing embryo^1^. These events encompass the block to polyspermy, completion of meiosis, maternal mRNA recruitment, and pronuclear formation^1^. Distinct cytoplasmic calcium transient patterns in the egg triggered by sperm-derived oocyte activating factors - such as phospholipase C (PLCζ) − upon fertilization dictate specific events of egg activation^2^. Interestingly, specific calcium transient profiles produced by the zygote are highly associated with embryo quality^3^,^4^,^5^,^6^. Moreover, altering the natural pattern of calcium transients in the zygote can have long-term effects on gene expression in the embryo and on offspring development^7^. Although calcium transient patterns in the egg are highly indicative of developmental outcomes, monitoring these transients requires intracellular imaging dyes and thereby prohibits the clinical application of this biological readout.

Recently, we and others have demonstrated in rodent, porcine, and nonhuman primate species that zinc is another essential element involved in meiosis and egg activation^8^,^9^,^10^,^11^,^12^,^13^,^14^,^15^,^16^,^17^,^18^. In mouse, meiotic maturation – or the progression of the gamete from a cell arrested in prophase of meiosis I into a mature egg arrested at metaphase of meiosis II - is accompanied by a substantial (50%) increase in total zinc content that is required for proper meiotic progression^11^. At fertilization, however, total zinc levels must decrease and within minutes of fertilization, zinc is released from the zygote in a secretory event termed the “zinc spark”^10^,^16^. Physiologically, this zinc release closely follows calcium transients and is necessary for cell cycle resumption via pathways that include modulation of the cell cycle regulatory protein EMI2^9^,^17^. Treatment of mouse eggs with a membrane-permeable zinc chelator reduces intracellular zinc availability and is sufficient to induce egg activation^10^,^11^,^17^. Moreover, live and fertile offspring can be obtained when diploid embryos generated by injecting inactivated sperm heads followed by zinc chelator treatment are transferred to recipient mice, and this takes place in the complete absence of calcium oscillations^17^. These results suggest that precise zinc regulation alone is sufficient to control events of egg activation and embryo development. Thus, a zygote’s zinc spark profile may be an early extracellular marker of its developmental potential, and modulation of zinc may be a valuable clinical tool.

Our understanding of the role of zinc during egg activation has been limited to studies in model organisms because the moments after human egg activation represent a privileged time with respect to basic science. The significant gaps in knowledge of the early events of development in our species arise from both the rare and unique nature of the human egg and from the ethical and legal restrictions surrounding studies involving parthenogenesis or fertilization^19^,^20^,^21^. We have demonstrated previously that human eggs contain zinc transporters and cortically enriched zinc vesicles, suggesting that zinc may have a prominent role during human egg activation^10^,^13^,^16^. The goal of this study was to extend our knowledge of the zinc spark from model organisms to human. Using three independent egg activation methods (calcium ionomycin, ionomycin, and hPLCζ cRNA), we define zinc flux as an inorganic signature of the human egg-to-embryo transition and demonstrate that chelation of intracellular zinc alone induces egg activation. These experiments not only further define the fundamental biology of human egg activation, but also lay the foundation for applying zinc flux clinically to decode the extracellular zinc spark profile as a readout of gamete potential and for developing zinc chelation as an activation method.

## Results

### Zinc sparks are associated with parthenogenetic activation

**Figure 1:**
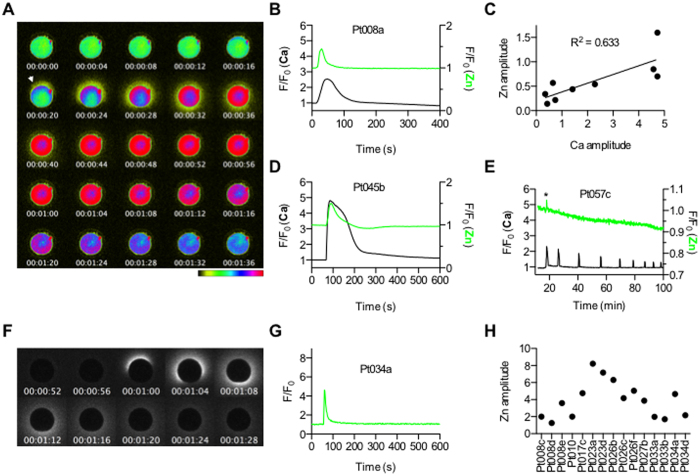
The zinc spark occurs in mature human eggs in concert with the rise in intracellular calcium in response to various egg activation methods. (**A**) A representative time course montage of calcium and zinc activation responses observed in a human MII egg following egg activation with 20 μM Ca-ionomycin (Pt008a). Intracellular calcium was monitored using 1 μM Fluo-4-AM and extracellular zinc was monitored using 50 μM FluoZin-3. The initial zinc spark signal is indicated by the white arrowhead. The LUT color scale is displayed at the bottom. (**B**) A time trace of normalized calcium (black) and zinc (green) fluorescence (F/F_0_) is shown for the montage. (**C**) Plot of Ca amplitude vs zinc amplitude in activated MII eggs (N = 9). Linear regression analysis indicates a positive correlation between the values (R^2^ = 0.633). (**D**) A Time trace of normalized calcium (black) and zinc (green) fluorescence (F/F_0_) following egg activation with 20 μM of ionomycin (apo form, no Ca) are shown (Pt045b). (**E**) A time trace of normalized calcium (black) and zinc (green) fluorescence (F/F_0_) following injection of hPLCζcRNA at 0.2 mg/ml (Pt057c) is shown. Two of the 5 eggs successfully activated with hPLCζ cRNA exhibited a zinc spark. An asterisk highlights the zinc spark that occurs concomitantly with the first calcium transient. (**F**) A representative time course montage of an MII egg activated with 20 μM Ca-ionomycin followed by only monitoring of extracellular zinc using 50 μM FluoZin-3 (Pt034a). (**G**) A time trace of normalized zinc fluorescence (F/F_0_) following egg activation with 20 μM of Ca-ionomycin is shown from Pt034a. (**H**) A plot of zinc amplitude in MII eggs activated with 20 μM Ca-ionomycin from a total of 8 different participants shows the variation in zinc sparks that is observed among eggs (N = 15).

In the mouse, activation of mature metaphase II-arrested (MII) eggs (herein referred to as eggs) either by fertilization or chemical means stimulates exocytosis of zinc from vesicles enriched at the egg cortex accounting for a loss of ~20% of the zinc content of the cell, and such zinc sparks have also been documented in two nonhuman primate species^10^,^16^. To examine whether zinc loss occurs during human egg activation, we first treated human eggs with the calcium ionophore, Ca-ionomycin, which delivers a bolus of exogenous calcium directly into the egg and bypasses the sperm-induced signaling cascades needed to elicit a rise in endogenous stores of intracellular calcium^22^. We then monitored intracellular calcium and extracellular zinc dynamics in individual eggs by live-cell imaging using Fluo-4-AM and FluoZin-3 dyes, respectively. Zinc was released from the periphery of the human egg coordinately with the rise in intracellular calcium within seconds of exposure to 20 μM Ca-ionomycin, (Fig. 1A,B, Movie S1). A positive correlation existed between the zinc spark and the calcium transient, with a larger intracellular calcium wave associated with a larger amplitude of zinc exocytosis (Fig. 1C). Moreover, we validated the egg activation-induced zinc spark using two additional approaches that stimulate the release of endogenous calcium stores, including ionomycin (apo form, without Ca) and microinjection of human PLCζ (hPLCζ) cRNA. The male specific PLCζ recapitulates the sperm induced Ca^2+^ oscillations by generating inositol 1,4,5-trisphosphate (IP_3_) from phosphatidylinostitol (4,5)-bisphosphate hydrolysis. This reaction supports the involvement of the IP_3_-mediated Ca^2+^ release from intracellular stores in mammalian fertilization^23^,^24^. These approaches elicited zinc sparks that were tightly coordinated with the first calcium transient (Fig. 1D,E). Importantly, the zinc spark was also observed when only zinc exocytosis was monitored with FluoZin-3 at the time of egg activation, indicating that the signal was not an artifact of the calcium imaging (Fig. 1F,G). Of note, there were differences in zinc spark amplitude profiles between individual eggs both among and between individual participants suggesting underlying differences in gamete quality (Fig. 1H). We are thus able to report that the zinc spark occurs during human egg activation and parallels the intracellular calcium transient.

### The acquisition of zinc spark potential is meiotic-stage dependent

**Figure 2:**
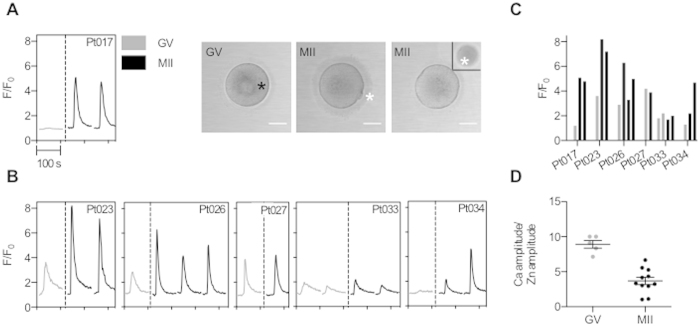
The acquisition of zinc spark potential is meiotic-stage dependent. (**A**) Time traces of normalized zinc fluorescence (F/F_0_) and transmitted light images of a prophase I-arrested GV oocyte and two metaphase II (MII)-arrested eggs from the same participant following activation with 20 μM Ca-ionomycin (Pt017). Extracellular zinc was monitored using 50 μM FluoZin-3. (**B**) Additional time traces of normalized zinc fluorescence (F/F_0_) and (**C**) the zinc spark amplitude in GV (grey) and MII (black) gametes activated with Ca-ionomycin is shown for 6 participant-matched samples (N = 7 GV oocytes and N = 12 MII eggs) (**C**) Comparison of the ratio of the Ca transient and zinc spark amplitude in GV (N = 5) and MII (N = 11) gametes show that MII eggs tend to have a lower Ca/Zn ratio compared to GV oocytes (t-test, p < 0.0001).

To determine whether the zinc spark was a hallmark of fertilization-competent gametes, we compared the zinc spark profiles of immature prophase I-arrested germinal vesicle-intact (GV) oocytes (herein referred to as oocytes) to those of mature eggs (Fig. 2, Movie S2). We first performed this comparison by monitoring extracellular zinc during Ca-ionomycin treatment with participant-matched samples (i.e. GV oocytes and MII eggs from the same individual) to minimize confounding variables. Using this approach, we found that the zinc spark amplitude was higher in MII eggs compared to GV oocytes in samples from 4 of the 6 participants (Fig. 2A–C). In a subset of GV oocytes and MII eggs from multiple participants, we monitored both intracellular calcium and extracellular zinc and compared the ratio of the calcium transient amplitude to the zinc spark amplitude (Fig. 2D). The ratio of the calcium:zinc fluorescence intensities provides a strong indication of how well a cell can respond to rises in intracellular calcium, with smaller ratios corresponding to more robust zinc responses. We found that MII eggs exhibited a smaller ratio on average compared to GV oocytes, indicating that zinc sparks are more prominent relative to intracellular calcium transients in MII eggs (Fig. 2D).

We further corroborated these findings in the mouse (Supplemental Fig. 1). We collected GV oocytes and MII eggs from gonadotropin-stimulated mice and monitored zinc exocytosis in response to ionomycin-induced egg activation in parallel (Supplemental Fig. 1). We found that, as was observed in human, there was a meiotic maturation dependence of the mouse gamete’s ability to elicit a zinc spark following parthenogenetic activation (Supplemental Fig. 1). Thus, across the species examined there was a strong correlation between meiotic stage and the zinc spark response, with gametes arrested at prophase I having on average a smaller zinc spark compared to those at the metaphase II stage.

### Intracellular zinc chelation is sufficient to induce human MII egg activation

**Figure 3:**
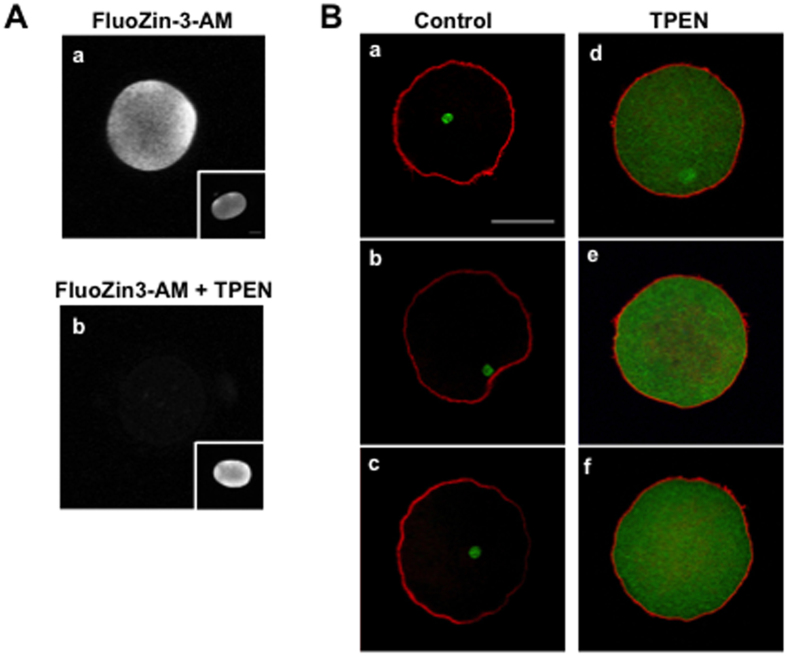
Zinc chelation is sufficient to induce human egg activation. (**A**) Intracellular labile Zn levels and localization were detected using Fluozin3-AM in MII eggs before (a) and after (b) treatment with 50 μM TPEN. Insets show intracellular labile zinc in control MII eggs treated with 0.5% DMSO. (**B**) Immunocytochemistry for cytoskeleton components (actin; red and tubulin; green) was performed following DMSO (a–c; N = 3) or TPEN treatment (d–f; N = 4). DMSO-treated eggs maintained their MII arrest as evidenced by intact spindles whereas TPEN-treated eggs had activated and completed meiosis as evidenced by an interphase tubulin network.

Three different methods of parthenogenetic activation (Ca-ionomycin, ionomycin, and hPLCζ cRNA) elicited zinc exocytosis, providing strong evidence that the zinc spark is a very early hallmark of egg activation. To further understand what the biological function of this zinc loss may be in the human, we mimicked activation-induced zinc loss by using the zinc-specific chelator, N,N,N′,N′-tetrakis(2-pyridylmethyl)ethane-1,2-diamine (TPEN) (Fig. 3)^11^. Treatment of MII eggs with 50 μM TPEN perturbed intracellular labile zinc, reducing it to undetectable levels as assessed by staining with the zinc-specific fluorophore Fluozin-3-AM (Fig. 3A). In comparison, labile zinc levels remained similar in DMSO-treated MII eggs (Fig. 3A, insets). We found that zinc insufficiency induced by TPEN alone resulted in MII egg activation. Within 3 hours, 100% of TPEN-treated human MII eggs had completed meiosis II and entered into the mitotic cell cycle as evidenced by the mesh-like appearance of the microtubule cytoskeleton network that is characteristic of mitotic interphase (Fig. 3B). In contrast, all DMSO-treated human MII eggs remained arrested in meiosis II as evidenced by the presence of the meiotic spindle (Fig. 3B). Thus this data suggests that lowering of intracellular zinc availability induces the cell cycle transition from meiosis to mitosis – an essential first step in the human MII egg-to-embryo transition or in this case, parthenogenesis.

## Discussion

Here we extend our previous work in the mouse and nonhuman primate and document, for the first time in humans, that zinc exocytosis is associated with egg activation and that a reduction in zinc is sufficient to trigger cell cycle resumption^10^,^11^. Due to legal restrictions on research with human eggs, we were limited to parthenogenetic activation methods to interrogate the zinc spark. Of the methods we used, injection of hPLCζ cRNA is the most physiologically relevant approach as it induces a series of calcium transients similar to sperm-induced activation^24^,^25^. This method produced zinc sparks, and in mouse we captured the sperm-mediated zinc spark in real time during *in vitro* fertilization. These results strongly support the physiological significance of this biological event (Zhang *et al.*, Sci Reports, accepted). During our studies in both mouse and human, we noted that there was a meiotic maturation-dependent acquisition of the ability of the gamete to mount a zinc spark, with cells arrested at prophase I having on average a smaller zinc spark compared to those arrested at metaphase II. These results suggest that the machinery that elicits the zinc spark is likely not fully established until just prior to fertilization, which may be an important mechanism to prevent premature egg activation. In the mouse, a significant increase of intracellular zinc occurs during meiotic maturation that is regulated predominantly by the zinc transporters ZIP6 and ZIP10^13^. Moreover, a prominent polarization of cortical vesicle distribution also occurs in the gamete between prophase I and metaphase II, and these vesicles are the source of the zinc spark^16^. Thus, there appears to be a spatio-temporal regulation of the gamete’s ability to produce a zinc spark, which is likely to be conserved as the human gamete both expresses ZIP6 and ZIP10 and has labile zinc localized to punctate vesicle-like structures^13^.

This tight coordination between the acquisition of zinc spark capacity and the timing of egg activation parallels what has been observed with cortical granule exocytosis (CGE) and calcium transients. For example, cortical granules are secretory structures that contain proteases that cleave Zona Pellucida Glycoprotein 2 and are involved in establishing the block to polyspermy. Gametes arrested at prophase I are less competent than mature MII eggs to undergo CGE, even when stimulated with treatments that induce calcium transients that mimic what occur during fertilization^26^. Both CGE and zinc exocytosis are calcium-dependent events, so our results are consistent with the knowledge that the egg’s ability to elicit repetitive calcium transients also increases throughout meiotic progression. GV oocytes, for example, are unable to initiate and maintain calcium transients as efficiently as MII eggs^27^,^28^. In response to ionomycin, mouse MII eggs exhibit a significantly greater calcium response compared to GV oocytes, and the greatest change was observed between metaphase of meiosis I and MII^29^. This differential calcium transient response is due to meiotic maturation-associated changes in calcium homeostasis mechanisms, including calcium channel modifications and densities, endoplasmic reticulum (ER) reorganization, and increases in ER calcium stores^30^. We therefore anticipate that the acquisition of zinc spark potential will mirror what has already been demonstrated for calcium^29^. However, future studies are warranted to define the precise timing during meiotic maturation when the gamete is able to achieve a maximal zinc spark response.

Although both mouse and human GV oocytes had a significantly reduced ability to produce a zinc spark in response to ionomycin activation relative to MII eggs, the response of mouse GV oocytes in general was more uniform and attenuated compared to the human GV oocytes. This discrepancy may reflect species-specific differences in calcium machinery and signaling mechanisms. For example, hamster GV oocytes already contain ~80% of the IP3-sensitive calcium stores present in MII eggs^31^. Alternatively, we can not discount the possibility that there may be inherent differences in the quality of the mouse and human gametes. For example, the mouse GV oocytes were obtained following gonadotropin stimulation protocols that enrich for a synchronous population of fully-grown oocytes, whereas the human GV oocytes were obtained because they failed to resume meiosis following hyperstimulation and are likely more varied in quality. This possibility underscores the inherent challenges of conducting basic research on human gametes – namely that the majority of the oocytes are those discarded from Assisted Reproduction Technologies (ART) cycles because they did not meet the developmental and/or morphological criteria for clinical use and that the sample size is limited. Despite these challenges, use of similar human oocyte populations for basic research has yielded important knowledge of human egg biology^32^,^33^,^34^.

The work described here, in addition to providing new insight into the fundamental biology of the human egg, also has important translational impact for ART in two distinct ways. First, intracytoplasmic sperm injection (ICSI), the process in which a sperm is microinjected directly into the egg, was originally developed to overcome male factor infertility but now accounts for up to roughly 70% of procedures done in clinical ART (www.sart.org). Although ICSI results in successful fertilization ~70% of the time, complete fertilization failure still occurs in up to 5% of the cases due to the inability of the egg to properly activate^25^. To improve the success of ICSI in patients with low fertilization potential, methods combining ICSI and egg activation have been implemented clinically. This technique, referred to as Assisted Oocyte Activation (AOA), relies on approaches that mimic or induce the fertilization-induced intracellular calcium rise such as calcium ionophore or PLCζ^23^,^25^. Successful pregnancies have been reported using AOA^35^. We demonstrated that treatment with an intracellular zinc chelator can activate human eggs, and thus may have important applications in AOA. In mouse, the fertilization-induced zinc spark, unlike calcium transients, is confined to the first minutes of egg activation (Zhang *et al.*, Sci Reports, accepted). Because of this narrow window, reducing intracellular zinc availability may be a more targeted and specific approach for driving egg activation, and may thereby increase the efficacy of AOA. Importantly, TPEN-mediated AOA has resulted in healthy live offspring in murine and porcine models^14^,^17^.

Second, advances in understanding the physicochemical roles of zinc in gamete function may have clinical impact because the zinc spark occurs rapidly following egg activation in the human egg and can be detected in the extracellular space. Moreover, there are variations in both the zinc spark and calcium transient profiles between individual eggs suggesting underlying differences in quality. In fact, elegant studies in the human have shown that calcium transient profiles following sperm-induced egg activation are distinct depending on egg quality parameters^4^. For example, processes known to diminish gamete quality, including *in vitro* maturation, extended culture, and vitrification and thawing, all result in alterations of the frequency and amplitude of calcium transients during fertilization^4^. In the human, establishing the direct relationship between zinc dynamics during fertilization and further embryonic development has not been possible. To address this important knowledge gap, we demonstrated in the mouse that parameters of the first zinc spark (amplitude and total zinc release) are highly associated with blastocysts of increased quality (Zhang *et al.*, Sci Reports, accepted). Efforts are currently underway to develop platforms to detect and quantify the human zinc spark in a non-invasive, safe, and efficacious manner. Specifically, methods by which exocytosed zinc in the media can be measured following fertilization are needed to limit the exposure of the zygote to both dyes and damaging photons required for imaging. The zinc spark technology possesses several advantages over existing embryo selection methods as this biological phenomenon occurs extracellularly within minutes of fertilization, and specific zinc spark profiles strongly correlate with and are predictive of preimplantation embryo development (Zhang *et al.*, Sci Reports, accepted). Thus, selecting embryos based on their fertilization-induced zinc spark profile would minimize the need for extended embryo culture and multiple embryo transfer – both of which have measureable risks^36^,^37^,^38^,^39^. Such changes would improve reproductive outcomes for ART, which now account for millions of births worldwide.

## Materials and Methods

### Human gamete acquisition

Female gametes used for egg activation experiments were obtained solely from the Fertility Center of Illinois (FCI), and all aspects of these egg activation studies were done in accordance with Northwestern University’s Institutional Review Board (IRB) guidelines and under IRB approval. We recruited participants undergoing intracytoplasmic sperm injection (ICSI) for scheduled infertility treatment, and following written informed consent, we collected immature female gametes that would otherwise have been discarded as routine practice. At FCI, only eggs that are at the MII stage within 2 hours of retrieval are used clinically for insemination, and all other immature cells are discarded. We obtained this discarded material in the research lab approximately 3–5 hours post-retrieval. A medical courier was used to transport immature oocytes from FCI to Northwestern University in a portable incubator maintained at 37 °C in Quinn’s Advantage Medium with HEPES supplemented with 10% Quinn’s Advantage Serum Protein Substitute (Origio, Trumball, CT).

Upon arrival to our research laboratory, gamete samples were labeled with the corresponding participant ID (Pt00X) and each gamete was assessed by light microscopy. Only morphologically normal cells that were at the correct meiotic stage were used for downstream experiments involving egg activation and live cell imaging. The sample size was therefore limited by the availability of participants that strictly met the selection criteria (female, ≥18 years old, English speaking, undergoing IVF-ICSI treatment for a non-cancerous diagnosis, and signing the informed consent document) and the quality of their gametes. Upon receipt, 33% of cells were arrested in prophase of meiosis I as evidenced by an intact germinal vesicle (GV), 30% had resumed meiosis but had not reached metaphase of meiosis II (MII), 22% had reached MII, and 15% were degenerate (Supplemental Fig. 2A). Cells were either used immediately or were *in vitro* matured further to the MII stage. Importantly, for experiments that required GV oocytes (Fig. 2), the cells at this meiotic stage were used immediately upon arrival and were not subjected to additional *in vitro* maturation (IVM). For IVM, gametes were placed in SAGE IVM medium (Origio) that had been pre-equilibrated in 4-well plates at 37 °C in 5% CO_2_. Cells that were in the GV-intact stage upon arrival took 28.0 ± 6.8 hours to reach MII, whereas those that had undergone GVBD already upon arrival only took 15.8 ± 9.7 hours. Typically it takes human oocyte 36–42 hours to reach MII following IVM^32^. Thus, based on this timing, the cells that we used in our study were largely meiotically-competent and had resumed meiosis while still in the ovary but were simply on the delayed end of the distribution. All cells used in this study had normal appearing cellular and subcellular morphology as assessed by cytoskeleton analysis (Supplemental Fig. 2B). Experimental treatments were tested in cells from a minimum of three separate participants. All data was included in this study as described.

### Animal care and welfare

Animals used in this study were handled following the National Research Council’s Animal Care and Welfare Guidelines. These procedures were approved by the Institutional Animal Care and Use Committee (IACUC) at Northwestern University. All the experiments were carried out in accordance with the approved guidelines.

### Chemicals and reagents

Fluorescent indicators (FluoZin-3, FluoZin-3-AM, Fluo-4-AM), ionomycin, Ca-ionomycin, and pluronic acid were obtained from Life Technologies (Grand Island, NY). Ammonium acetate, ionomycin, Ca-ionomycin and other chemicals were purchased from Sigma Aldrich (St. Louis, MO). Ionomycin and Ca-ionomycin were stored as single-use aliquots in DMSO at −20 °C. TPEN was dissolved in DMSO immediately before each use and was diluted into buffer such that buffers contained ≤0.5% DMSO. Paraformaldehyde was purchased from Electron Microscopy Sciences (Hatfield, PA).

### Live cell fluorescence microscopy of MII egg activation by ionophore

In Zn-only imaging experiments, single gametes were placed on one end of a long shallow 19 μL drop of 50 μM FluoZin-3 (for Zn monitoring) in Ca-free hCZB medium under oil in a coverslip-bottom imaging dish. In experiments in which intracellular Ca and extracellular Zn were monitored, gametes were first incubated in 1 μM Fluo4-AM (for Ca monitoring) and 0.02% Pluronic F-127 in SAGE oocyte washing medium (Origio) for 30 minutes at 37 °C^40^. Gametes were then washed through dye-free medium and placed in 50 μM FluoZin-3 in Ca-free hCZB as described above. Imaging was performed at 37 °C on a TCS SP5 confocal microscope using 488 nm excitation and an open pinhole (Leica Microsystems, Buffalo Grove, IL). Initial fluorescence images were obtained prior to activation. For activation imaging, Ca-ionomycin (1 μL) was introduced to the imaging drop on the end opposite that of the gamete to yield a final concentration of 20 μM. Imaging was initiated immediately following Ca-ionomycin addition. To induce egg activation through endogenous calcium stores, 20 μM Ionomycin (apo form, without Ca) was added to the imaging drop as described above. Images were collected every 2 seconds for a minimum of 5 minutes as described above. Image analysis was performed by defining regions of interest (ROIs) and measuring fluorescence intensity over time using ImageJ^41^. Intracellular ROIs were defined as the entire interior area of the cell. Extracellular ROIs were defined as a ring around the perimeter of the cell. The ring thickness was conserved for all data analyses.

### Live cell fluorescence microscopy of MII egg activation by hPLCζ cRNA microinjection

The pBluescript RN3 plasmid containing the full-length coding sequence of human PLCζ (generous gift from Dr. Rafael Fissore, University of Massachusetts-Amherst) was linearized at a site beyond the 3′ end of the coding sequence, and *in vitro* transcribed using the T3 mMessage mMachine kit (Ambion, Austin, TX). mRNA was purified according to manufacturer’s instructions. Prior to microinjection, concentrated cRNA (2 mg/ml) was heated for 3 min at 85 °C to reduce secondary structure and diluted as needed in nuclease free water. hPLCζ cRNA was microinjected in the cytoplasm of human eggs final concentrations of 0.05 mg/ml and 0.2 mg/ml. For microinjections, human eggs were first incubated in 1μM Fluo4-AM and 0.02% Pluronic F-127 in SAGE oocyte washing medium (Origio) for 30 minutes at 37 °C. Eggs were then microinjected with hPLCζ cRNA. For microinjection, human MII eggs were transferred to drops of L-15 medium containing 0.05% (w/v) polyvinyl alcohol and 0.1% (w/v) sucrose (Invitrogen) under light mineral oil on a heated stage set to 37 °C. hPLCζ cRNA was back-loaded into glass micropipettes and delivered by a XenoWorks Digital Microinjector (Sutter Instrument, Novato, CA). The injection volume was ~28–48 pl (1–3% of the total volume of the egg). The eggs were then washed through dye-free medium and placed in 50 μM FluoZin-3 in hCZB as described above.

### Zinc chelation and labile zinc analysis

To analyze whether TPEN treatment perturbed intracellular zinc content, human eggs were first loaded with 10 μM Fluozin3-AM supplemented with 0.02% pluronic acid for 30 min at room temperature and then the eggs were incubated in CZB medium (Millipore, Temecula, CA) containing 0.5% DMSO with or without 50 μM TPEN for 30 min at 37 °C in an atmosphere of 5% CO_2_ in air. The eggs were then imaged at 37 °C on a TCS SP5 confocal microscope using 488 nm excitation and an open pinhole (Leica Microsystems, Buffalo Grove, IL). To determine the effect of zinc chelation on cell cycle progression, human MII eggs were incubated in CZB medium (Millipore, Temecula, CA) containing 0.1% PVA with or without 50 μM TPEN and cultured for 2 hours at 37 °C in an atmosphere of 5% CO_2_ in air. TPEN was freshly prepared in DMSO at stock concentration of 10 mM. Control medium contained 0.5% DMSO to achieve the same DMSO concentration as in TPEN drops. Eggs were then transferred to KSOM medium (Millipore) and cultured up to an additional 3 hours. Extended culture following parthenogenetic activation was not possible due to specifications in our IRB protocol. Following culture, the cells were fixed and processed for immunofluorescence as described below to confirm cell cycle stage.

### Immunofluorescence and microscopy

For cytoskeletal characterization (actin and tubulin), cells were fixed in 3.8% PFA 0.1% Triton-X in PBS at 37 °C for 1h. Cells were transferred to blocking buffer (0.01% Tween 20, 0.01% NaN_3_, 3 mg/mL BSA, PBS) and stored at 4 °C prior to staining. Samples were permeabilized in 0.1% Triton-X in PBS for 15 minutes at room temperature, washed in blocking buffer, and incubated in 1:100 tubulin-FITC (Cell Signaling, Beverly, MA) and 1:50 rhodamine phalloidin (Life Technologies) overnight at 4 °C. Samples were then washed 3 × 30 minutes in blocking buffer and mounted on slides using Vectashield^®^ (Vector Labs, Burlingame, CA). FITC and rhodamine fluorescence was detected using a TCS SP5 confocal microscope (Leica) using 488 nm and 543 nm laser excitation respectively.

### Statistical analysis

Zinc spark response in immature and mature gametes was compared using a two-tailed unpaired t-test. Statistical tests were performed using the software Prism 5.0 (GraphPad). P < 0.05 was considered statistically significant.

## Additional Information

**How to cite this article**: Duncan, F. E. *et al.* The zinc spark is an inorganic signature of human egg activation. *Sci. Rep.* **6**, 24737; doi: 10.1038/srep24737 (2016).

## Supplementary Material

Supplementary Movie S1

Supplementary Movie S2

Supplementary Information
