# Supplementary material for: The zinc spark is an inorganic signature of human egg activation
Source: Sci Rep. 2016 Apr 26;6:24737. doi: 10.1038/srep24737 (PMC4845039; doi:10.1038/srep24737)

The zinc spark is an inorganic signature of human egg activation

**Authors:** Francesca E. Duncan^1†^, Emily L. Que^2†^, Nan Zhang^1†^, Eve C. Feinberg^3^, Thomas V. O’Halloran^2,4*^, Teresa K. Woodruff^1*^

**Supplementary Materials:**

*Gamete collection*

To collect MII eggs, CD-1 female mice, 6–8 weeks old, were injected with 5 IU of pregnant mare serum gonadotropin (PMSG; Sigma, St. Louis, MO). MII eggs were obtained from the oviducts 12–14 h after injection of 5 IU hCG, which was administered 46–48 h after PMSG injection. Eggs were released into HEPES-buffered tyrode-lactate solution (TL-HEPES) supplemented with 5% heat-treated fetal calf serum (FCS; Gibco BRL, Grand Island, NY) followed by treatment with 0.1% bovine testes hyaluronidase for 3–5 min to remove cumulus cells. MII eggs were then thoroughly washed and transferred into 50 ml drops of KSOM (Potassium Simplex Optimized Medium; Specialty Media, Phillipsburg, NJ) containing 0.1% BSA under paraffin oil at 36.5°C and in a humidified atmosphere containing 5% CO_2_ before imaging. GV oocytes were obtained from the ovaries 44 h post-PMSG in TL-HEPES supplemented with 5% heat-treated FCS and 10 μM milrinone. GV oocytes were also thoroughly washed and transferred into 50 ml drops of KSOM containing 0.1% BSA and 10 μM milrinone under paraffin oil at 36.5°C and in a humidified atmosphere containing 5% CO_2_ before imaging.

*Zinc spark imaging and analysis*

Mouse MII eggs and GV oocytes were placed in a 360µl drop of 50 µM Fluozin-3 (for zinc monitoring) in Ca-free hCZB medium under oil in a coverslip-bottom imaging dish (MatTek Corp, Ashland, MA). Imaging was performed at 37 °C on a TCS SP5 (Leica) confocal microscope (Leica Microsystems, Heidelberg, Germany) equipped with a stage top incubator (ToKaiHit, Shizuoka, Japan), 20x objective and an Ar (488nm) laser line. Initial fluorescence images were obtained prior to activation. A 40µl 10x stock solution of 50µM Ionomycin was introduced to the imaging drop 1 minute after the start of zinc imaging. Images were collected every 4 seconds for 10 minutes. Image analysis was performed by defining regions of interest (ROIs) and measuring fluorescence intensity over time using ImageJ. Extracellular ROIs were defined as a ring around the perimeter of the cell. The ring thickness was conserved for all data analyses.

*Immunofluorescence and microscopy*

For cytoskeletal characterization (actin and tubulin), cells were fixed in 3.8% PFA 0.1% Triton-X in PBS at 37°C for 1h. Cells were transferred to blocking buffer (0.01% Tween 20, 0.01% NaN_3_, 3 mg/mL BSA, PBS) and stored at 4 °C prior to staining. Samples were permeabilized in 0.1% Triton-X in PBS for 15 minutes at room temperature, washed in blocking buffer, and incubated in 1:100 tubulin-FITC (Cell Signaling, Beverly, MA) and 1:50 rhodamine phalloidin (Life Technologies) overnight at 4 °C. Samples were then wash 3x30 minutes in blocking buffer and mounted on slides using Vectashield® (Vector Labs, Burlingame, CA). FITC and rhodamine fluorescence was detected using a TCS SP5 confocal microscope (Leica) using 488nm and 543nm laser excitation respectively.

**Supplementary Figures**

**Figure S1. Meiotic maturation dependence of the zinc spark in the mouse**

(A) A representative montage of the mouse zinc spark response following activation in prophase I-arrested GV oocytes (cells 6-9) and metaphase II-arrested eggs (cells 1-5). Extracellular zinc was monitored using Fluozin-3. Representative time traces of the zinc spark amplitude based on the normalized zinc fluorescence are shown of (B) a GV oocyte and (C) MII egg. (D) A comparison of the zinc spark amplitude among all gametes examined shows that MII eggs have a significantly higher zinc spark response compared to GV oocytes (t-test; P < 0.001). This experiment was repeated three times and a total of > 35 cells at each meiotic stage were examined.

**Figure S2. Additional characteristics of the human gametes used for research.**

(A) Meiotic progression of gametes used for research was scored by morphology upon arrival to the research laboratory within a few hours of retrieval. Immature cells were arrested at prophase of meiosis I as evidenced by an intact nucleus or germinal vesicle (GV) or had resumed meiosis and undergone germinal vesicle breakdown (GVBD) but had not reached metaphase of meiosis II (MII). Mature eggs had reached MII as evidenced by polar body extrusion. These cells were either used immediately or in vitro matured further to obtain cells at the correct stage of meiosis. (B). Human gametes appeared healthy based on their cytoskeleton morphology assessed by actin (red) and tubulin (green) staining. Representative confocal optical sections are shown. Note the characteristic interphase microtubule network (GV), bipolar spindle (MII), and cortical actin. Scale bars 50 µM.

**Supplementary Files**

Movie S1. Ca-ionomycin-induced Ca transient and zinc spark (Pt008)

Move S2. Zinc-only imaging of Ca-ionomycin treatment of a GV oocyte compared to participant-matched MII eggs (Pt017).


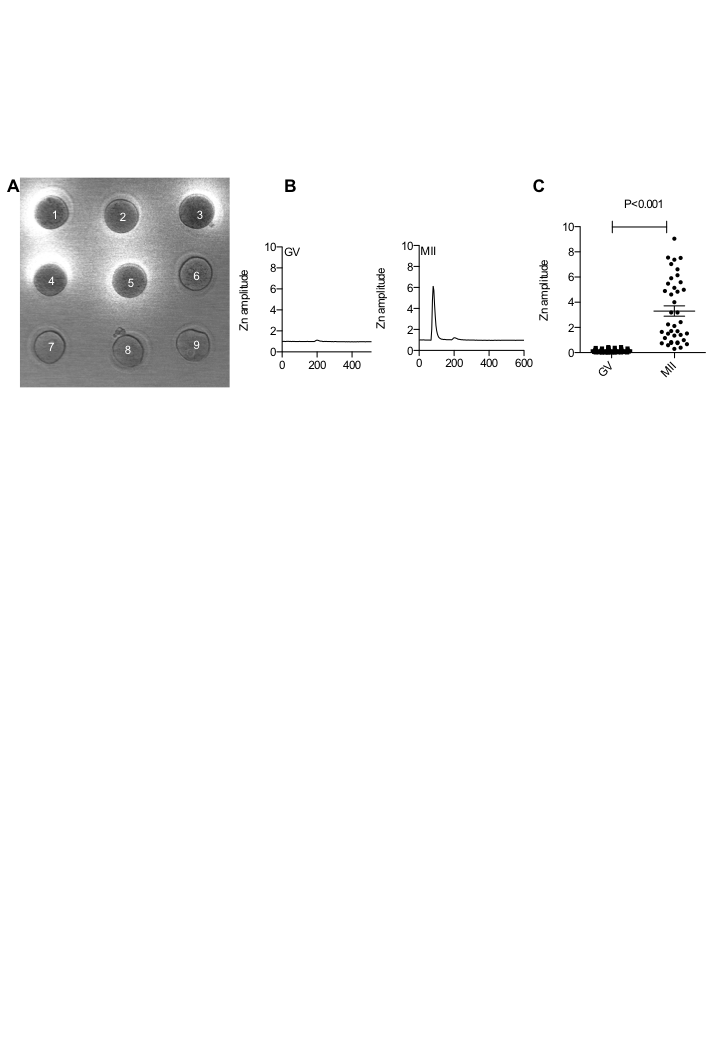


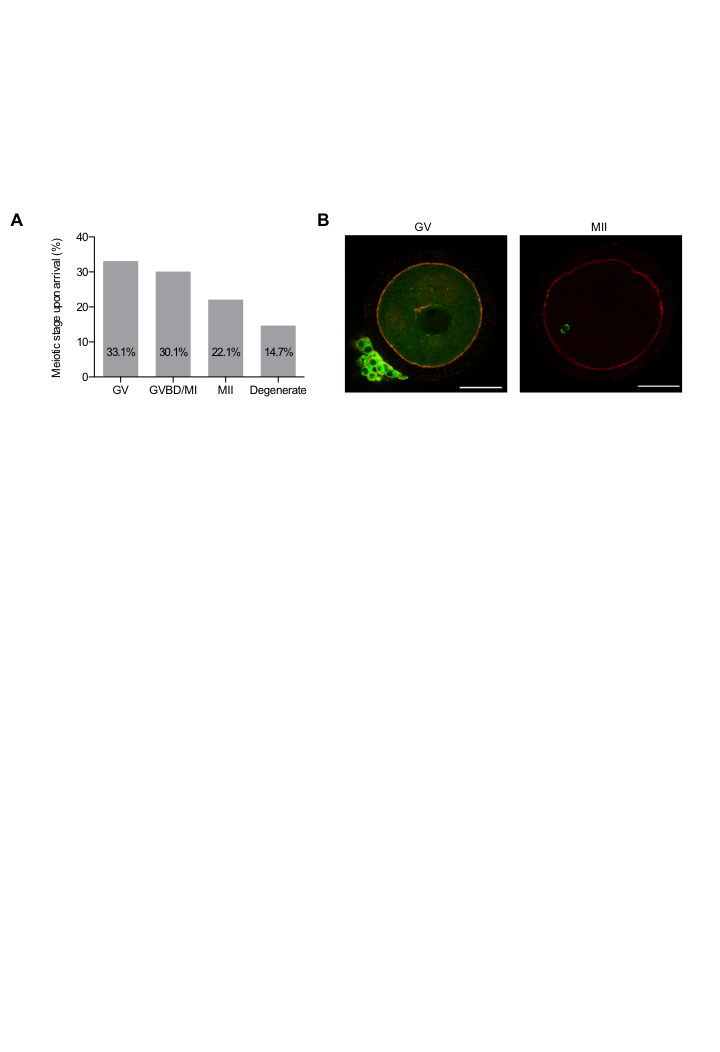

Supplement: Supplementary Information [file srep24737-s3.docx]
